# Perspective: Advancing spectator behavior research in youth sports through a closer examination of racial differences

**DOI:** 10.3389/fspor.2022.933472

**Published:** 2022-08-11

**Authors:** Jerry F. Reynolds, Cassandra D. Chaney, Olivia Huffman

**Affiliations:** ^1^Department of Social Work, Ball State University, Muncie, IN, United States; ^2^School of Social Work, Louisiana State University, Baton Rouge, LA, United States; ^3^Department of Sport and Exercise Psychology, Ball State University, Muncie, IN, United States

**Keywords:** youth sports, critical race theory, implicit bias, spectator behavior, policing of youth sports

## Abstract

Inappropriate spectator behaviors are a recognized challenge within both amateur and youth sport settings. These behaviors occur during youth sports contests and involve several sources of interaction, and impact the experience of child athletes, coaches, parents, and referees Spectator misconduct reflects a failure to self-regulate amidst disagreement with the coaching practices, officials, and poor performance from children. Despite widespread recognition of spectator misconduct and an emphasis by the United States Department of Health and Human Services (DHHS) to improve parent behavior, limited empirical research is available to promote understanding of both contributors to these actions, more specifically, what parents are observing from others and the frequency of such behaviors. A path to enhance research in this area is a closer examination of intersectionality, especially race and its influence upon parent observations and their personal behaviors as youth sport spectators. Based on research conducted in Louisiana, this perspective piece reflects on a study that found race as a contributing factor to differences in spectating behaviors of parents. The authors unpack the nuances of these findings through a lens of both Critical Race Theory (CRT) and implicit bias and provide a platform for future study, especially in states such as Louisiana where laws and the role of police have been advanced to mitigate spectator behaviors in youth sport settings.

## Discussion of current advances

This article aims to share some advances in parent spectator behavior research in youth sports settings. These advances involve scholarship concerning new laws designed to create legal consequences and an expanded role for law enforcement to address these challenges. Inappropriate spectator behaviors can be characterized as common, impulsive, and with limited legal consequences (Fields et al., 2007; Goldstein and Iso-Ahola, 2008; Walters et al., 2016; Block and Lesneskie, 2018). Negative spectator behaviors (often represented by parent misconduct) predict negative athletic behaviors of children and reduced enjoyment for all participants (Arthur-Banning et al., 2009; Bean et al., 2014; Logan and Cuff, 2019).

In recent years, The National Federation of High School Sports (NFHSS) has contended spectator behavior misconduct has reached epidemic proportions (Niehoff and Bonine, 2019). In addition, spectator misconduct has become so problematic that according to data from the National Association of Sports Officials (2017), contended spectator behavior was responsible for the mass exodus of referees across sports settings. While there are frequent mass media and social media stories about these incidents, there is limited empirical evidence to understand the frequency and nature of spectator behaviors (Goldstein and Iso-Ahola, 2008; Omli and Lavoi, 2009; Knight, 2019), especially when laws are created to legislate such behaviors.

To contextualize the link between laws and spectator behaviors, the authors used existing tools and researched perceptions of spectator behaviors within 6 months of a law passed to a new law in the state of Louisiana. Omli and Lavoi (2009) provided a foundation for research in this area. They surveyed parents in 2009 and developed a 10-item, five-point Likert scale survey through which they captured parent experiences as spectators. Behaviors on this survey list included examining parent interactions with other spectators, children, and their personal activities. The scales had appropriate levels of reliability in both the Omli and Lavoi (2009) study and the discussed study (Reynolds, 2020). This study used a similar scale, with minor modifications to items to be more descriptive and appropriately capture spectator experiences.

## Louisiana context of spectator behavior challenges

While there was not a shortage of concern about spectator behaviors nationally, Act 355 was passed in Louisiana to expand both what constituted inappropriate actions and the role of law enforcement in such settings. The impetus for this study was to examine how legislation shapes parent behaviors and the negative publicity associated with these actions (LHSAA, 2019a,b).

The legislation extended the scope of spectator laws to define verbal and physically abusive activities that were previously limited to the assault on referees. Act 355 called for the expansion of the role of police in a state already challenged by public acts of police brutality that captured national attention in both the years preceding and after the passage of Act 355. Notable crimes included the public deaths of the African-American men, Alton Sterling (on 5 July 2016) and Ronald Greene (on 10 May 2019) at the hands of White police officers and ongoing assertions that Blacks are less likely to be treated fairly police and the use of excessive force fostered a tense-policing environment in the state. Added to this is the history of racism in sports (Davis, 2007). Given that such a large portion of youth both in the state and nationally participate in sports and publicly display their behaviors as spectators, it is important to look at spectator behaviors as another avenue through which both Blacks and Whites have contact with the police and report their observations to law enforcement. Important to consider is how race influences parents to modify their behaviors not only to avoid contact with police but also due to fear of injustice and or retaliation.

Act 355 impacts all the sanctioned athletic events (competitive and recreational) in the state and outlined consequences for verbal and physical abuse of all the participants, both on and off the field. The law prohibits behaviors (both verbal and non-verbal) that potentially place an individual in harm's way because of experiencing physical or verbal abuse (Act 355). The legislation obligates schools and youth sports organizations to enact additional safety protocols (LHSAA, 2019a,b). Violators are subject to fines, jail time, and community service (LHSAA, 2019a,b).

The purpose of this expanded discussion is to connect differences in reporting personal and observed youth sports spectator behavior in the context of new legislation (i.e., Act 355) to mitigate maladaptive spectator behavior in a tense-policing environment. This piece provides a lens into the reporting behaviors of parents when regulating others and considers the impact of such legislation on referees and parents of minoritized demographic backgrounds. Furthermore, we posit that differences in spectator behaviors are informed by race. These differences should be considered when creating legislation for other states. There are two reasons why our study is unique: 1) We examined parents perceptions of their personal spectator behaviors and those they observed from others in the state within 6 months after the passage of Act 355 and 2) We explored how variables such as race influenced parent behaviors (both personal and observed).

## Method

In the Spring of 2019, the lead author of the study became aware of Act 355 and its signing into law in August of 2019. Upon receiving IRB approval from the sponsoring institution, the author used an adapted version of Omli and Lavoi (2009) 10-item questionnaire concerning spectator behaviors to survey parent spectators (ages 18–64 years) whose children (ages 6–18 years) had participated in a team sport in the state of Louisiana and resided in the state during the past year. The same 10 items were used for both observed and personal behaviors, and both were measured on a 5-point Likert scale, with responses ranging from (1) Never to (5) All of the time. Upon completion of the survey, the 10 items were totaled, resulting in an aggregate score of both observed and personal behaviors that ranged from a low of 10 to a maximum score of 50. A score of 10 represented parents who reported “Never” personally participating in behaviors listed on the survey. A score of 10 on the observed scale meant the participant never observed such activities at a youth-sporting event.

Snowball sampling strategies *via* social media recruited participants during the months of December 2019–February 2020. From recruitment, 106 youth sports spectators (75% women) participated in this study with 23.8% of participants African-American or Black (73.6% White and 2.8% Asian or Pacific Islander). Demographic information about the individual's race was collected. The participant's city of residence or geographic location were not collected in the survey to preserve confidentiality. In addition to the spectator behavior (observed and personal) items, participants were asked about their awareness and knowledge of Act 355, received a nine-slide educational PowerPoint presentation regarding Act 355, and completed Omli and Lavoi (2009) adapted survey pre- and post-PowerPoint presentation. Results were analyzed using *t-*test and repeated measures ANOVA. This perspective expands the discussion from the Reynolds (2020) study.

## Results

Reynolds (2020) used the 10-item adapted Likert scale from Omli and Lavoi (2009), which results displayed differences among those surveyed (*N* = 106) specifically between the observed and personal spectators of Whites and minority youth sports parents. White participants who identified as parents of a youth sports athlete (*n* = 78) in the study on average reported a 10-point total difference across all the items between their observations of spectators and their personal behaviors, whereas African-Americans and other minorities (*n* = 28) members reported a smaller difference between these two variables.

### Differences in reported behaviors

**Figure 1:**
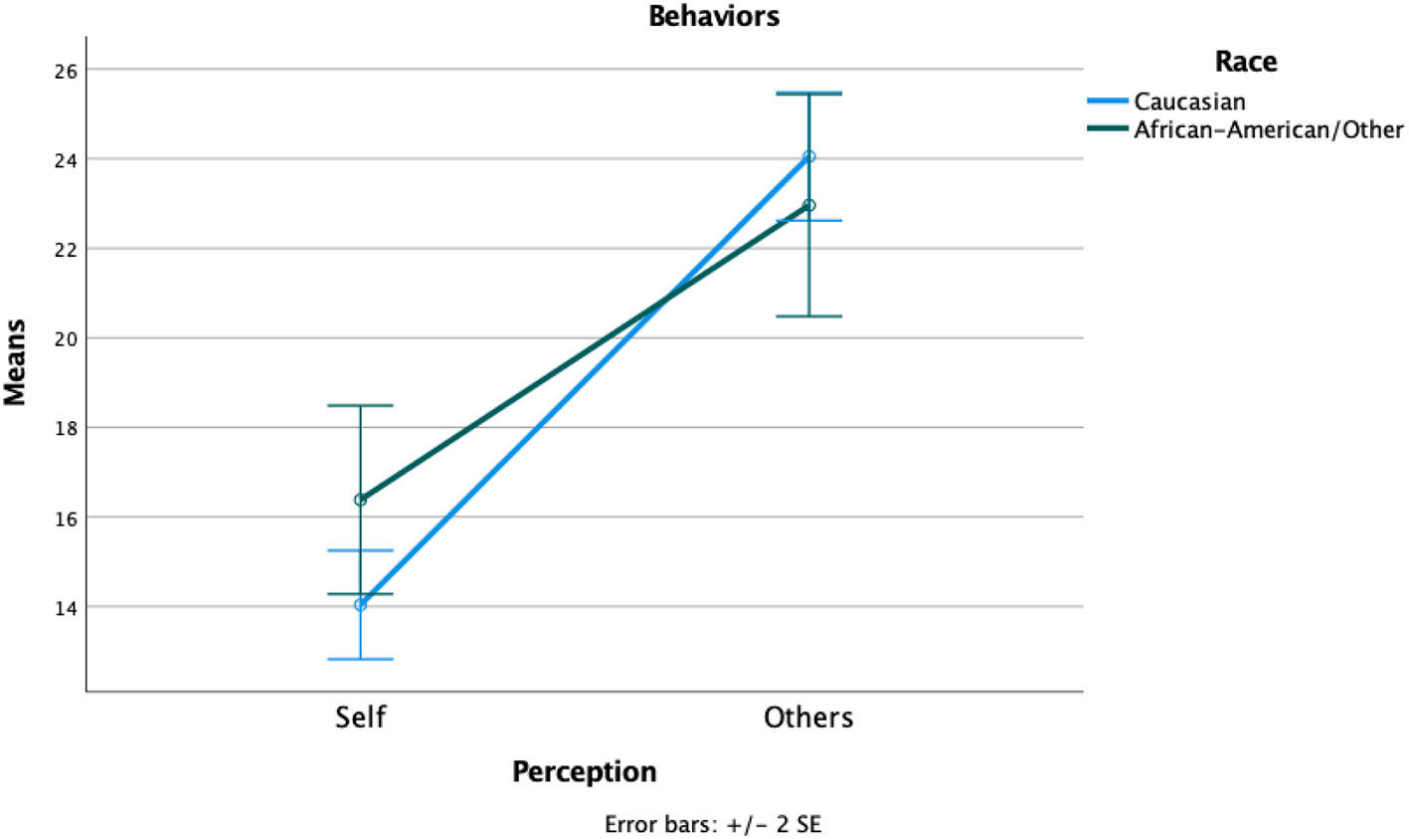
Spectator behavior means comparison by identified race.

Across Reynolds' (2020) sample, and the 10-item Likert scale (1 to 5 points) in which participants assessed the frequency of behaviors (personal and observed). Scores ranged from 10 to 50 points. Overall, observed behavior revealed a mean score of 23.78 (SD = 6.03), and a large difference from personal behaviors (*M* = 14.62, SD = 5.44). When broken down by the identified race, there were notable differences in personal scores for the Caucasian parents (*M* = 14.04, SD = 4.12) and those who identified as members of minority groups (*M* = 16.38, SD = 8.07). Observed behavior scores also demonstrated statistically significant differences by race with the Caucasian parents (*M* = 24.05, SD = 5.439) and minority parents (*M* = 22.96, SD = 8.483; Figure 1). There was less of a gap in differences between observed and personal behaviors of minority participants who prompts future research to delve more into these associated nuances.

## Author perspective

While it is a part of human nature to have different experiences, this study provided a unique context to examine spectator behaviors. The state of Louisiana provides an important context for approaching spectator behaviors, especially, as Act 355 emerged during a tense-policing environment and widespread social unrest. There are several manifest contextual factors to consider with regard to this legislation, especially as law enforcement increases interactions with spectator behaviors. Other factors to consider include but are not limited to (a) the structure of police departments, (b) reporting differences between blacks and whites, (c) perceptions of police, and the theoretical perspectives of both implicit bias and critical race theory.

## Structure of police departments

Act 355 places the responsibility of enforcement upon local police departments. Like most police departments in the country (Leatherby and Oppel, 2020), the majority of police in Louisiana are White and men (Mustian, 2018). After Mississippi, Louisiana, at 32.8% has the country's second-highest proportion of the African-American residents, (Mustian, 2018). In light of the growing national and local attention on law enforcement's excessive force on Black bodies and disproportionate arrest rates (Haynes, 2020; Srikanth, 2020). Several scholars have asserted White officers target Black people, because they inherently perceive them as more dangerous and criminal than other races (Weitzer and Tuch, 1999; Nix et al., 2017; Robertson and Chaney, 2019; Jackson-Jefferson, 2020). Thus, it is important to examine the circumstances by which Blacks and Whites have contact with, initiate contact with police, and report their observations to law enforcement. In Louisiana, this is now more possible, given the heightened expectation and role of law enforcement in addressing spectator behaviors in youth sports settings.

## Reporting differences between blacks and whites in Louisiana

Our study revealed race plays a role in how parent spectators perceive both their observed and personal spectator behaviors. Data from the Bureau of Justice Statistics' 2015 Police–Public Contact Survey (PPCS), a supplement to the National Crime Victimization Survey (NCVS) is especially informative. Whites were more likely than Blacks, Hispanics, and persons of other races to contact police to report a crime, a non-crime emergency, or to seek help for some other reason (Davis et al., 2018). Since most police officers in the nation are White (Najdowski et al., 2015), it is logical to assume that White men would be more likely to initiate contact with police than Black men. Thus, it is essential to determine how Blacks and Whites view police, particularly concerning perceptions that police will treat them fairly, especially in sport-based settings where sportsmanship and fairness are valued.

## Comparisons between how blacks and white view police

There are other contextual factors to consider, especially related to the perceptions of police, who now have an enhanced role in enforcing laws and norms related to spectator behaviors. Compared to Whites, African-Americans generally have less confidence that the police will treat them fairly (Tyler, 2005; Brunson, 2007; McLeod et al., 2020). One noteworthy study found found that when compared with Whites, Blacks were approximately twice as likely as Whites to believe the police do not have valid reasons to stop people, that police are too tough on people they stop, and that police are verbally or physically abusive toward citizens. Nearly, 30% of Blacks hold these views, as compared with 11–15% of Whites (Weitzer et al., 2008).

In a setting such as youth sports, where fairness and good sportsmanship are expected, it is important to consider perceptions of fair treatment by police. Recent data indicate that African-Americans have little trust that police will treat them fairly. The findings of a 2019 Pew Research Center Survey revealed that 84% of Black adults said they are “generally treated less fairly than Whites,” while 63% of Whites had this view. Similarly, “87% of Blacks and 61% of Whites said the U.S. criminal justice system treats black people less fairly. Black adults are about five times as likely as whites to say they have been unfairly stopped by police because of their race or ethnicity (44 vs. 9%).” While there is much to be explored concerning the dynamics of these findings, it warrants viewing these findings through two important theoretical frameworks. Future research must consider these dynamics within spectator behaviors, especially when there are notable differences in findings and expectations of police to enforce such laws.

## Implicit bias

The sports setting is one in which allegiance to one's team and support of their own child is paramount to parents. This also extends to those perceived as different or “the opponent.” As such, humans generally perceive members of their own racial group more favorably than they generally perceive those who are not members of their group. In contrast to explicit bias, which is conscious, overt demonstrations of racial prejudice, implicit bias operates from an unconscious level and allows individuals to assess everyday circumstances and categorize the individuals with whom they come in contact. Implicit bias is not necessarily a bad thing. As Lawson (2015) wrote, “Implicit bias can be found in good people of every racial background. The tendency for police officers to view their own actions in the best light possible, or to shade or stretch the truth to protect their personal interests, reflects the human condition.” (p. 344). The propensity to perceive members of one's own group positively is intrinsic to the human condition. Lawson (2015) explained, “One reason for such associations is that we humans are wired to view our own groups as superior to others and to exaggerate differences between our own group and outsiders.” (p. 348). Since implicit associations about social groups influence every aspect of our environment (e.g., family, schools, television, newspapers, and movies), continued exposure to racial stereotypes inevitably leads to implicit associations and implicit racial bias. An example of an implicit racial bias is the propensity for law enforcement officers to assume that a Black man at a sporting event is more likely to behave erratically at a sporting event than a White male. Since the police are sworn “to protect and serve” all individuals in society, implicit racist bias may invariably motivate them to treat civilians differently (Chaney and Robertson, 2013). How can the implicit racial bias of police affect African Americans? Essentially, although ingrained, individuals can consciously decide against allowing racial biases to determine their actions (Plant and Peruche, 2005).

Police implicit racial bias can cause African-Americans to fear being judged by the public and to demonstrate certain behaviors to minimize these fears. Najdowski et al. (2015) conducted research that investigated how Blacks experience encounters with police officers, in particular, whether such encounters induce Blacks to feel stereotype threat, or concern police will judge them and treat them unfairly. As predicted, Black men, but not Whites, reported concern that police officers stereotype them as criminals simply because of their race. In addition, Black but not White men anticipated feeling stereotype threat in the hypothetical police encounter, which involved experiencing more anxiety and regulating their behavior. Police officers frequently perceive regulated behaviors as suspicious.

Since the negative stereotype of criminality can cause Blacks to feel police will negatively judge them and treat them unfairly (Najdowski et al., 2015), it makes sense that Blacks may feel this way in various environments. Our findings suggest this could extend to the youth sports setting and may suggest that African-Americans may modify their behavior to avoid contact with police.

## Critical race theory

Race is a social construct, and humans have used this construct to socially advantage some individuals and disadvantage others (Morning, 2007). Since race is embedded within various systems (i.e., educational, governmental, penal, sports), one must acknowledge how race may determine how Blacks and Whites behave in certain settings. Sport is also a domain embedded with a history of racism, exclusion, and exacerbation of white privilege (Davis, 2007). Critical race theory (CRT) is an academic framework that places race in the center of all human interactions, recognizing that how people perceive and conduct themselves is inextricably linked to race (Chaney and Robertson, 2013). Thus, CRT acknowledges racism is more than mere demonstrations by individual people with prejudices but is a systemic issue that recognizes how the legal system disadvantages minorities. CRT recognizes the various experiences of people of different races. Essentially, CRT acknowledges that while members of some races can avoid police scrutiny, members of other races are targets of police. This suggests African Americans may self-regulate, intentionally behaving in ways that decrease the likelihood Whites will perceive them negatively during sporting events and thus avoid contact with members of law enforcement, who are generally White males.

## Shaping future research

This study makes important contributions to the literature, especially concerning how race shapes parent perceptions of both their personal spectators and those observed in youth sports settings. As Block and Lesneskie (2018) explain, documenting the nature of spectator behaviors is complex and limited studies have documented the frequency and nature of spectator behaviors. Reynolds (2020) affirms previous research from Omli and Lavoi (2009) concerning frequent behaviors of parents and suggests that race is a variable in shaping perspectives on spectator behaviors. Furthermore, as both legislators and the youth sports environment grapple with how to develop laws to protect spectators from harm and create a civil youth sports environment, they must consider nuances that shape enforcement of laws and policing practices. They must consider the historical challenges associated with the racial tensions within both the sports environment and broader society, along with the biases one may bring to reporting incidents of inappropriate behavior. In addition, it is important that scholars examine these intricacies, particularly regarding how they both shape how parents perceive their behaviors and laws and various factors that influence policing in youth sport settings.

## Data availability statement

The raw data supporting the conclusions of this article will be made available by the authors, without undue reservation.

## Ethics statement

The studies involving human participants were reviewed and approved by Louisiana State University Institutional Review Board. The patients/participants provided their written informed consent to participate in this study.

## Author contributions

JR is the lead author on the project. JR completed the research for the article and oversaw the development of and execution of the manuscript. CC was JR's dissertation advisor and she made many of the contributions related to critical race theory and implicit bias in the manuscript. OH helped craft the introduction, the theory application within this article and the data analysis. All authors contributed to the article and approved the submitted version.

## Conflict of interest

The authors declare that the research was conducted in the absence of any commercial or financial relationships that could be construed as a potential conflict of interest.

## Publisher's note

All claims expressed in this article are solely those of the authors and do not necessarily represent those of their affiliated organizations, or those of the publisher, the editors and the reviewers. Any product that may be evaluated in this article, or claim that may be made by its manufacturer, is not guaranteed or endorsed by the publisher.
